# A New Case of *de novo* Variant c.892C>T (p.Arg298Trp) in NACC1: A First Case Report From China

**DOI:** 10.3389/fped.2021.754261

**Published:** 2021-11-15

**Authors:** Baiyu Lyu, Yan Dong, Juan Kang

**Affiliations:** Department of Pediatrics, Shanghai Ninth People's Hospital, Shanghai Jiaotong University School of Medicine, Shanghai, China

**Keywords:** NACC1, intellectual disability, infantile epilepsy, congenital cataract, feeding difficulties

## Abstract

**Background:** The nucleus accumbens associated 1 (NACC1) gene is a transcription factor member of the BTB/POZ family. A *de novo* heterozygous c.892C>T (p.Arg298Trp) variant in the NACC1 may define a syndrome characterized by intellectual disability, infantile epilepsy, congenital cataract, and feeding difficulties.

**Case Presentation:** We report a new case with a neurodevelopmental disorder characterized by severe intellectual disability, infantile epilepsy, congenital cataract, and feeding difficulties. Brain MRI reveals brain dysplasia. We observe a *de novo* heterozygous c.892C>T (p.Arg298Trp) variant in the NACC1 gene in this case. Now, the child regularly goes to the hospital for rehabilitation training (once a month). Sodium Valproate (10 mg/kg/day) and Clobazam (10 mg/kg/day) are used in the treatment of epilepsy. A total of three articles were screened, and two papers were excluded. The search revealed one article related to a syndrome caused by a *de novo* heterozygous c.892C>T (p.Arg298Trp) variant in the NACC1; they screened the main clinical features of eight cases of a syndrome, which were summarized and analyzed.

**Conclusions:** The NACC1 gene is a member of the BTB/POZ family of transcription factors. A *de novo* heterozygous c.892C>T (p.Arg298Trp) variant in the NACC1 may define a syndrome characterized by intellectual disability, infantile epilepsy, congenital cataract, and feeding difficulties. At present, there is no effective cure. In the future, we need more cases to determine the phenotype–genotype correlation of NACC1 variants. Many questions remain to be answered, and many challenges remain to be faced. Future transcriptional studies may further clarify this rare, recurrent variant, and could potentially lead to targeted therapies.

## Introduction

NACC1 gene is a member of the BTB/POZ family of transcription factors (1). Many studies have found that NACC1 is highly expressed in various tumors (2). The highly expressed NACC1 is closely related to cell viability, migration, and tumor recurrence (3, 4). It has also been identified as a BTB gene associated with cancer. It has been reported that the NACC1 gene is associated with intellectual disability. Some studies suggest that the NACCI is a genome-wide significant disease-associated gene.

The prevalence of intellectual disability (ID) in newborns is 2–3% (5). In most cases, the cause is unknown (5), while 0.5% suffer from severe intellectual disability (ID), which is mostly genetically related (6). Congenital cataract (CC) refers to the genetic abnormality caused by lens opacity at birth. The prevalence of CC in every 10,000 children is 2.2–13.6 (7). Schoch et al. reported that the NACC1 gene mutations are related to a syndrome characterized by epilepsy, cataracts, feedings difficulties, and neurodevelopmental disorders (1). Although the gene coverage is relatively high, the single loss of function and the lower-than-expected number of missense variants indicates that mutations in NACC1 are affected by selection and are very rare. The predicted pathogenic variants may increase human disease risks (1). We will review the literature and report that a new case on the NACC1 c.892C>T (p.Arg298Trp) variant causes a syndrome. This article aims to expand the knowledge on phenotypic features and introduce the biological role of NACC1. This is the first case report from China and the eighth so far.

## Clinical Report

The patient was a 4-year-old Chinese Han female of *de novo* variant c.892C>T (p.Arg298Trp) in the NACC1 that causes a syndrome characterized by severe intellectual disability, infantile epilepsy, congenital cataract, and feeding difficulties. The patient was born at 37^+3^ weeks of gestation to non-consanguineous Chinese parents with one healthy twin boy and no family history of epilepsy or disability syndromes. She was born, and the pregnancy was uneventful. She was admitted to the neonatal intensive care unit on the third day of life for neonatal hyperbilirubinemia, with good recovery.

When she was 3 months old, she could not stand her head upright, and a white substance was visible in front of the pupil of the left eye, which moved with the eyeball. Ophthalmology was diagnosed as a congenital cataract.

At 7 months of age, the girl still could not stand her head upright and turn over. She could chase sounds and laughs when people tease her, and she has difficulty feeding (only bottle feeding, and easy to choke). Also, at 7 months of age, she suffered from her first seizure characterized by eye blinking, cyanosis around the mouth, and ankylosis of limbs. EEG findings show infantile epilepsy.

At 2 years of age, on clinical observation, she was behind her peers in intellectual development. She was diagnosed with intellectual disability by Wechsler Intelligence Scale for Children Fourth Edition-Chinese. Brain MRI showed widening frontal and temporal sulci and widening the subarachnoid space in the frontal and temporal regions. The volume of the white matter in the center of the semioval was small, and the corpus callosum was thin. At the same time, as rehabilitation training, levetiracetam (10 mg/kg/day) therapy was used for epilepsy.

**Figure 1:**
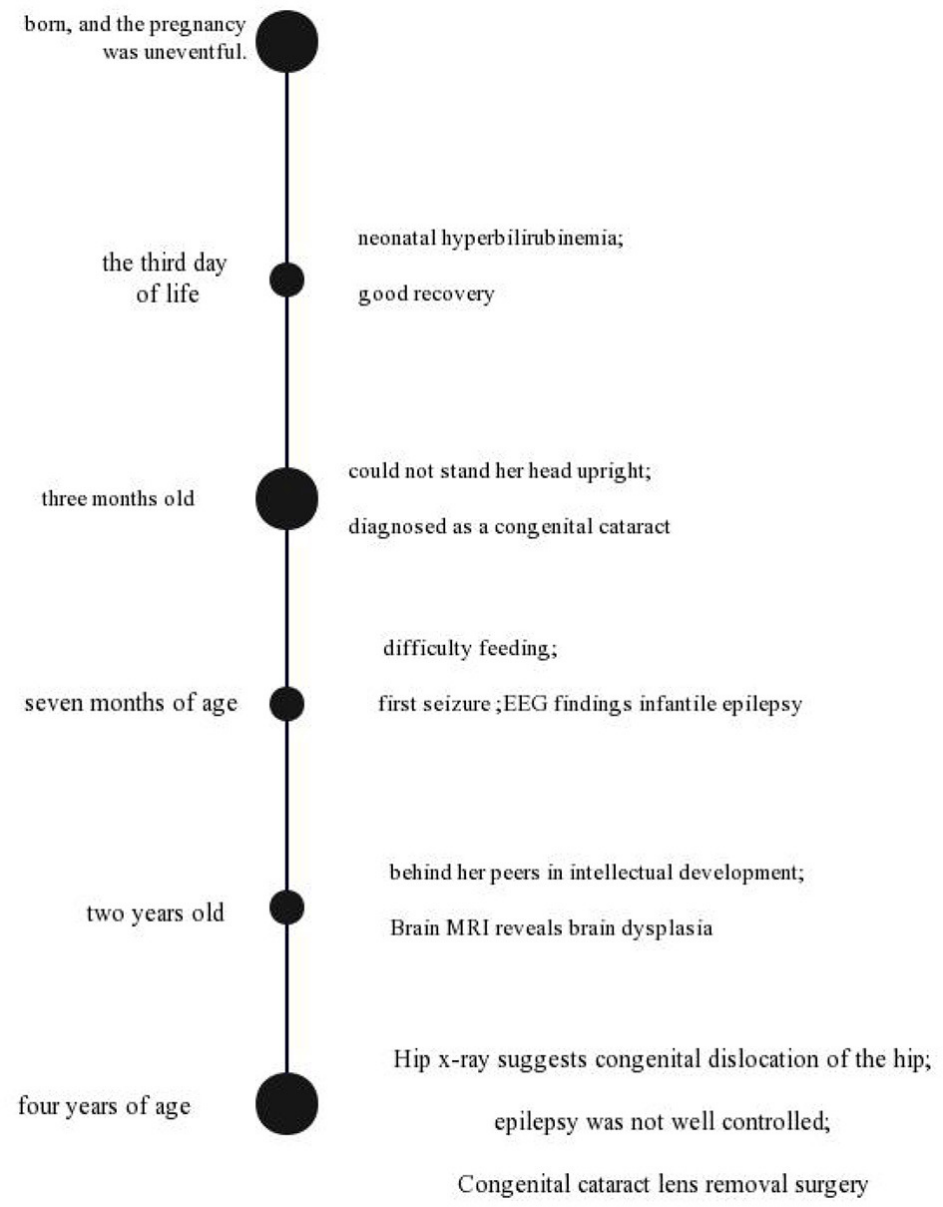
A timeline with relevant data from the patient episode of care.

When she was 4 years old, She could turn over, but she could not sit alone and walk and talk. Hip x-ray suggests congenital dislocation of the hip. At the same time, epilepsy was not well-controlled. Now, Sodium Valproate (10 mg/kg/day) and Clobazam (10 mg/kg/day) are used in the treatment of epilepsy. Congenital cataract lens removal surgery was carried out when the child was 4 years old. A timeline with relevant data from the patient episode of care is shown in Figure 1.

## Diagnostic Assessment

**Figure 2:**
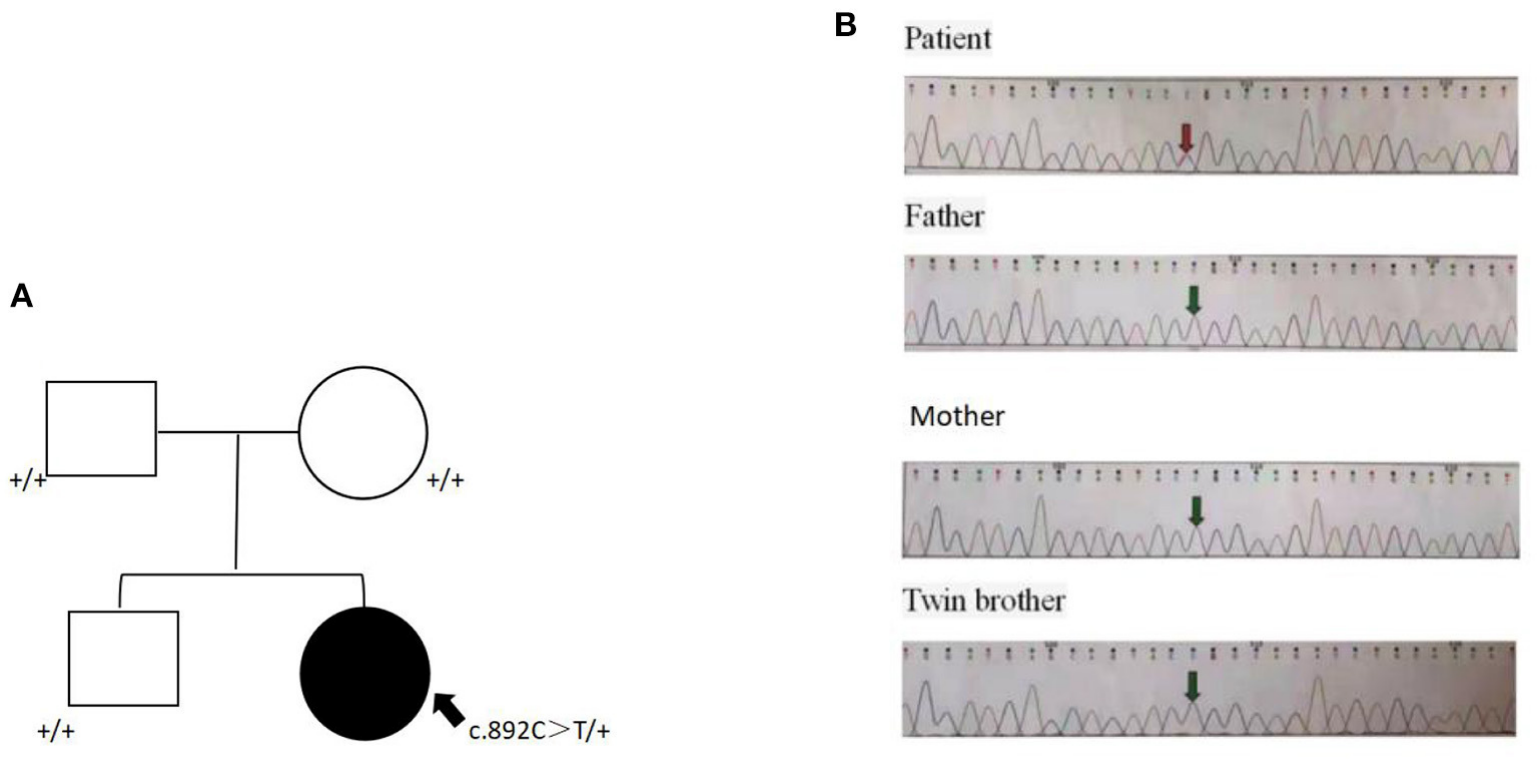
*De novo* c.892C>T (p.Arg298Trp) missense change in NACC1. **(A)** Pedigree of the family. A heterozygous missense was detected in the proband but not the parents. **(B)** Sanger validation of the missense variant in the proband and parents. Arrow indicates the site of the variant.

To confirm the diagnosis, sequencing was performed with the consent of the family members. Whole exon sequencing of the girl and parents and twin brother showed that the child was *de novo* heterozygous c.892C>T (p.Arg298Trp) variant in the NACC1 (GenBank NM_052876_3), as shown in Figure 2. Now, the child regularly goes to the hospital for rehabilitation training (once a month). Sodium Valproate (10 mg/kg/day) and Clobazam (10 mg/kg/day) are used in the treatment of epilepsy.

## Literature Review

Using “NACC1” and “intellectual disability” as keywords, the Chinese and English databases (PubMed, CNKI, Wanfang, and VIP databases) were searched for papers published up to July 15, 2021. A total of three articles were screened. Two papers (8, 9) were excluded because of cases without infantile epilepsy, congenital cataract, and feeding difficulties. The critical phenotypic features of all patients with NACC1 c.892C>T (p.Arg298Trp) variant published to date are summarized in Table 1.

**Table 1:** Phenotypic features of all patients with NACC1 c.892C>T (p.Arg298Trp) variant published to date.

	**Patient 1**	**Schoch K's (seven cases)**
Gender	Female	Five male; two female
Postnatal microcephaly	No	Five yes; two no
NACC1 c.892C>T (p.Arg298Trp) variant	*De novo*	*De novo*
Intellectual/developmental disability	Severe	Six profound; one severe
Seizures	Infantile epilepsy	Seven infantile epilepsy; four infantile spasms
Cataract	Left cataract	Five bilateral cataract; two no
Feeding difficulties	Yes	Yes
Hypotonia	Present	Six present; one absent
Stereotypic movements	Yes	Six yes; one no
Congenital dislocation of the hip	Yes	Unknown
Irritability	Yes	Yes
Brain MRI findings	Brain dysplasia	Six decreased brain volume; four delayed myelinations; one focal cortical dysplasia
Ref	This report	(1)

## Discussion

In this case, a *de novo* heterozygous c.892C>T (p.Arg298Trp) variant in the NACC1 causes a severe intellectual disability, infantile epilepsy, congenital cataract, profound developmental delays, feeding difficulties, and congenital dislocation of the hip. Previously reported microduplication/microdeletion syndrome involving chromosome 19p13.13 includes most NACC1 genes (10). Previous research has shown that mosaic mutations frequently occur in individuals diagnosed with autism spectrum disorders and intellectual disability (11). One individual with intellectual disability (IQ of ~45), schizo-affective disorder, and autism has been noted with a *de novo* missense allele c.1402C>T (p.Arg468Cys) in NACC1 (6). Another patient with autism spectrum disorder was distinguished to have a *de novo* splicing variant (probably LoF), c.946þ2T>C, in NACC1 (12).

Many studies have found that the NACC1 is highly expressed in a variety of tumors, such as ovarian cancer (3, 13), cervical cancer (3, 4), endometrial cancer (3, 14), breast cancer, renal cell cancer, pancreatic cancer (15, 16), and melanoma (17). Highly expressed NACC1 goes hand in hand with cell proliferation, migration and invasion, chemotherapy resistance, tumor recurrence, and poor prognosis (4). NACC1 knockout mouse embryos and newborns have lower survival rates (18).

Nucleus accumbens-associated protein 1 (NAC1) is encoded by the NACC1 gene (15) and is a member of the BTB/POZ protein family (19). BTB/POZ proteins can recruit histone deacetylases (20), ubiquitin-ligases (21), and corepressors (22) to construct complexes that modulate gene expression and typically modify chromatin conformation (19).

NAC1 was initially identified and cloned as a cocaine-inducible transcript from the nucleus accumbens, a unique forebrain structure involved in addictive behavior and reward motivation (15). Subsequently, NAC1 has been shown to play critical roles in a variety of biological processes, including pathogenesis of human cancer (23), the proliferation of embryonic stem cells, maintenance of stemness (24), participation in the psychomotor response of rats after taking cocaine (25), and targeting substrates to cullin-based E3 ligases for ubiquitin-dependent proteasome degradation (26). NAC1 plays an essential role in the pluripotency of embryonic stem cells (27) through direct transcriptional regulation of c-Myc (28). Furthermore, It has recently been demonstrated that NAC1 represses neuroectodermal fate selection and promotes mesendodermal in embryonic stem cells, in concert with the pluripotency transcription factors, Tcf3, Oct4, and Sox2 (15, 29). NAC1 is essential to generate iPSC (15). NAC1 is also a necessary part of RIG-I-like receptor-mediated innate immune responses against viral infection (19).

NAC1 plays a vital role in normal neurologic function by maintaining synaptic plasticity and reducing protein turnover in dendritic cells (1, 30). As a transcriptional repressor, NAC1 regulates cell growth, senescence, epithelial–mesenchymal transition, and autophagy. NAC1 prevents cytotoxicity by stabilizing hypoxia-inducible factor-1α and regulating glucose levels, thereby playing a role in the hypoxic tumor environment (31). NAC1 forms a homodimer or heterodimers with other binding partners through the BTB/POZ domain and exerts transcriptional inhibition through the recruitment of histone deacetylase (32). It has also been predicted that the BEN domain participates in chromatin organization and transcriptional regulation by mediating protein–DNA and protein–protein interactions (33). Although most of the functions of NAC1 are related to transcriptional regulation, it is also associated with cytokinesis through the NACC-1/actin/profilin-1 complex and the post-translational role of the protein in cancer cells (28, 31).

The c. 892C> T transition occurs at a CpG dinucleotide within an arginine codon, a highly variable CpG pattern associated with *de novo* events at many loci in setting advanced paternal age. However, advanced paternal age was not a common feature of this group (1). The p.Arg298Trp variant is located outside the BTB/POZ domain that is important for cancer progression. However, it is unknown whether individuals with the p.Arg298Trp variant are at risk for cancer (1). It has been reported that a missense variant located in the BEN domain is associated with intellectual disability (6). Because of the characteristic phenotype related to the p.Arg298Trp variant, future transcriptional studies may further clarify this rare, recurrent variant, potentially leading to targeted therapies.

The number of cases is still limited, many questions remain to be answered, and many challenges remain to be faced. In this case, after the diagnosis, the patient's families were very depressed when they learned that it is a rare disease for which there is no effective cure at present. However, they did not give up hope. They believe that future medical advancement will find a way to cure the disease.

## Data Availability Statement

The datasets for this article are not publicly available due to concerns regarding participant/patient anonymity. Requests to access the datasets should be directed to the corresponding author.

## Ethics Statement

The studies involving human participants were reviewed and approved by Ethics Committee of Shanghai Ninth People's Hospital, Shanghai Jiaotong University School of Medicine. Written informed consent for participation was not required for this study in accordance with the national legislation and the institutional requirements. Written informed consent was obtained from the legal guardian for publication of this case report and any accompanying images.

## Author Contributions

BL and YD conceived and supervised the study. BL analyzed data. BL and JK wrote the manuscript. BL, YD, and JK made manuscript revisions. All authors approved the final version of the manuscript.

## Conflict of Interest

The authors declare that the research was conducted in the absence of any commercial or financial relationships that could be construed as a potential conflict of interest.

## Publisher's Note

All claims expressed in this article are solely those of the authors and do not necessarily represent those of their affiliated organizations, or those of the publisher, the editors and the reviewers. Any product that may be evaluated in this article, or claim that may be made by its manufacturer, is not guaranteed or endorsed by the publisher.
